# Home and Wild Food Procurement Were Associated with Greater Intake of Fruits and Vegetables During the COVID-19 Pandemic in Northern New England in a Cross-Sectional Study

**DOI:** 10.3390/nu17101627

**Published:** 2025-05-09

**Authors:** Ashley C. McCarthy, Ashleigh Angle, Sam Bliss, Farryl Bertmann, Emily H. Belarmino, Kelsey Rose, Meredith T. Niles

**Affiliations:** 1 Department of Nutrition and Food Sciences, University of Vermont, 109 Carrigan Drive, 256 MLS Carrigan Wing, Burlington, VT 05405, USA; ashley.mccarthy@uvm.edu (A.C.M.);; 2 Gund Institute for Environment, University of Vermont, 210 Colchester Ave, Burlington, VT 05405, USA; 3 Food Systems Program, University of Vermont, Burlington, VT 05405, USA

**Keywords:** self-provisioning, gardening, hunting, fishing, fruit and vegetable intake, diet quality, meat intake, food insecurity, food system resilience, food sovereignty

## Abstract

**Background:** Participation in home and wild food procurement (HWFP) activities (i.e., gardening, hunting, fishing, foraging, preserving food, raising livestock, and raising poultry for eggs) skyrocketed during the COVID-19 pandemic. Procuring food through HWFP activities may have important food security and nutrition benefits, while also enhancing food sovereignty and food system resilience. This cross-sectional study examined the effect of HWFP activities on food security status, fruit and vegetable intake, and meat consumption. **Methods:** We used data collected in 2021 and 2022 from adults (*n* = 2001) through two statewide representative surveys in Maine and Vermont, United States. Dietary intake was assessed using the Dietary Screener Questionnaire. Food security status was assessed using the U.S. Department of Agriculture’s 6-item short-form food security module. We analyzed the data using linear regression, logistic regression, and ordinal logistic regression models. **Results:** Sixty-one percent of respondents engaged in HWFP activities; the majority of those gardened. Households engaging in most individual HWFP activities had greater odds of being food insecure. HWFP engagement was positively associated with fruit and vegetable consumption. Specifically, gardening was associated with an additional one cup-equivalent in fruit and vegetable consumption per week compared to respondents that did not garden. Furthermore, when exploring these relationships disaggregated by food security status, we find that this effect is stronger for food insecure households than food secure households. Respondents from households that hunted were more likely to eat wild game meat and also consumed red and white meat more frequently compared to households that did not hunt. **Conclusions:** Overall, our results indicate potential nutrition and food security benefits from engaging in HWFP activities. Future research should continue to examine a full suite of HWFP activities and their relationship to diet, health, food security, and food sovereignty.

## 1. Introduction

Engagement in home and wild food procurement (HWFP) skyrocketed during the COVID-19 pandemic. For example, in the early days of the pandemic, authorities in 41 of 47 surveyed U.S. states reported increased license sales for spring turkey hunting [1]. Participation in recreational fishing swelled too, in part because of the perceived safety of this outdoor activity; some anglers started to call it “social fish-tancing” [2,3]. Vegetable seeds and mason jar lids became hard to find as new and existing gardeners bought them in preparation for augmenting their production and preservation capacities [4]. Research shows people upped their participation in all these endeavors in part because they had more free time and wanted relief from the stress of the pandemic but also because they were worried about having enough food [5].

Procuring food through HWFP activities may have important food security and nutrition benefits while also enhancing food sovereignty and food system resilience. By growing and wild-harvesting their own food, individuals build knowledge and skills and may gain greater control of their food supply through improved access to sufficient, nutritious, and/or culturally appropriate foods, which are important aspects of food security and food sovereignty [6,7]. Furthermore, self-provisioning can help individuals build resilience to food system disruptions by diversifying food sources and reducing dependence on commercial food sources.

Growing and wild-harvesting one’s own food can serve as coping mechanisms for dealing with food insecurity [8,9]. A 2020 survey of Vermonters found that food insecure households were more likely to engage in a suite of HWFP activities except gardening in the first six months of the pandemic as compared to food secure households [10]. Furthermore, there is some evidence that these practices work to improve food security; among households that experienced food insecurity in the early months of the COVID-19 pandemic, those that engaged in HWFP were significantly more likely to become food secure a year into the pandemic [11].

Evidence of HWFP’s ability to improve diet and nutrition during the pandemic is less established. Across high-income societies, many studies have found an association between gardening and greater fruit and vegetable intake [12], though this effect has been identified in some cases as only occurring for food secure households [10]. However, many of these studies do not have non-gardening control groups [13,14,15,16], or they are correlational, precluding examination of causal relationships [17,18]. It may not be that households eat more vegetables because they garden, but the other way around; they garden because they like eating vegetables. A recent randomized control trial found that the group that received a gardening intervention increased their vegetable consumption compared to the control group, by 0.67 servings per day [19]. In interviews, gardeners said they ate more vegetables because of increased availability, better taste, trying new dishes, pride in their homegrown food, not wanting to waste it, and emotional connections with gardens and their plants [19].

Across high income countries, research on the dietary impacts of HWFP activities is limited to studies on gardening and associations between fishing and omega-3 fatty acid levels [20,21]. Rigorous research relating hunting, foraging, fishing, and raising livestock to the intake of relevant foods and nutrients comes entirely from the Global South or remote indigenous communities [22,23,24,25,26]. The few studies that examine a full suite of HWFP activities as a unified category [27,28,29] do not address diet, nutrition, or food security.

This study fills these gaps by exploring a suite of HWFP activities in a high-income country and looks at the relationships between HWFP activities and food security and dietary intake. We use survey data from a representative sample (*n* = 2001) of residents in Maine and Vermont, the two U.S. states with the greatest share of residents living in rural areas, to assess how household-level HWFP engagement relates to food security status and intake of foods that certain HWFP activities produce: fruits, vegetables, and game meat. Thus, our analysis goes beyond gardening to get a population-level view of the dietary implications of most HWFP activities. Unlike available studies on gardening and vegetable intake, we explore the differential effect that engaging in HWFP may have for food secure versus insecure households. Previous research has demonstrated that food security status can influence the potential benefits accrued through engagement in these activities [10,30].

We evaluate the following hypotheses:

**H1.**  *Engagement in HWFP is positively associated with food insecurity.*

**H2a.**  *Engagement in gardening, foraging, and preserving food are associated with higher fruit and vegetable intake*.

**H2b.**  *Food insecure households that gardened, foraged, and preserved food will have lower fruit and vegetable consumption, as compared to food secure households that engaged in these activities.*

**H3a.**  *Engagement in hunting is associated with eating game meat more frequently.*

**H3b.**  *Engagement in hunting is associated with eating red meat and white meat less frequently.*

**H3c.**  *Food insecure households that hunted will have greater odds of eating game meat, as compared to food insecure households that did not hunt.*

By gaining a fuller understanding of HWFP’s relationship with food security and diet outcomes, we hope to identify the potential of HWFP as a strategy to improve food security and diet quality.

## 2. Materials and Methods

### 2.1. Survey Development and Sampling Strategy

Data were collected through two surveys in the US states of Maine and Vermont. The first survey was conducted from March to June 2021 (*n* = 988), and the second survey was conducted from April to May 2022 (*n* = 1013). The survey instrument was initially developed in March 2020 by the National Food Access and COVID research team [31] and then expanded for 2021 and 2022 to include more questions about HWFP activities [32]. The survey included sections on food sourcing, HWFP, food security, diet intake, health outcomes and behaviors, experiences during the COVID-19 pandemic, and demographic information. Institutional Review Board approval was obtained prior to data collection. Participants were recruited through Qualtrics (Provo, UT, USA) research panels and completed an online survey. Qualtrics is a professional survey company that recruits participants from their proprietary research panels using a random sample. We used sample quotas in the sampling process to ensure our respondents were representative of the racial and ethnic distributions of the states based on the population profiles from the American Community Survey [33]. Once a quota category was filled, no additional respondents from the demographic group were able to participate. To be eligible to participate in the survey, participants had to be 18 years or older and live in the US states of Maine or Vermont. Respondents were anonymous in the data collection process.

### 2.2. Variables for Analysis

We used four categories of variables in our analyses: food security status, engagement in HWFP activities, dietary intake, and demographic information (Table 1).

Food security status was measured using the US Department of Agriculture 6-item short-form food security module [34]. Respondents were asked to assess whether their households had sufficient economic access to food during the 12 months prior to taking the survey. Following the standard protocol for calculating food insecurity, respondents who responded affirmatively to two or more questions were classified as food insecure.

Engagement in HWFP activities was assessed through a series of questions about whether anyone in the respondent’s household had gardened, hunted, fished, foraged, raised livestock for meat or milk, raised poultry for eggs, or preserved food during the 12 months prior to taking the survey. We analyzed engagement in any HWFP activity and engagement in specific activities.

Dietary intake was measured using the Dietary Screener Questionnaire (DSQ), a standardized and validated instrument to identify the frequency of intake of selected foods during the previous 30 days [35]. For each of the 26 items included in the screener, respondents chose one of nine frequency options ranging from never to multiple times per day. We added a question to the screener to measure frequency of game meat (e.g., venison, wild turkey or duck, pheasant, bear, etc.) consumption, since game meat is not included in the DSQ. Although the DSQ does not measure quantity consumed, scoring algorithms provided by the National Cancer Institute were used to convert screener responses to estimate daily intake (in cup equivalents or cup-eq) for fruits and vegetables following the established procedures [36]. Our analyses use these estimates of fruit and vegetable consumption rather than precise measurements of intake. Red meat, white meat, and game meat are reported on a consumption frequency basis and as a binary variable indicating absence or presence of any consumption.

Individual demographics include age, gender identity, race, ethnicity, and education level. Household demographics included annual household income, job disruption during the COVID-19 pandemic, and zip code. We used respondent zip codes and the Rural–Urban Commuting Area code system to classify respondent geography as urban or rural [37,38].

### 2.3. Statistical Analysis

To examine the relationship between HWFP engagement and food security status, we used logistic regression models, reporting the odds ratios. We constructed separate models for each individual HWFP activity, as well as a combined model that included all activities to understand interactions between them.

To examine the relationship between HWFP engagement and fruit and vegetable intake, we used linear regression models, reporting the coefficients. In these analyses, we looked at the three HWFP activities that produce fruits and vegetables: gardening, foraging, and preserving food. Once again, we used separate models that looked at each individual activity’s relationship to fruit and vegetable consumption, plus a combined model that included all three activities to understand any interactions between them. We also examined the relationship to food security status by running separate analyses only including food secure households and only including food insecure households.

To examine the relationship between hunting and frequency of meat consumption, we used logistic regression models for game meat and ordinal logistic models for red meat and white meat, reporting odds ratios. Due to the distribution of the data, we had to analyze game meat consumption as a binary variable (any consumption vs. no consumption) rather than looking at a more detailed range in consumption frequency. We also examined the relationship by food security status by running separate analyses only including food secure households and only including food insecure households.

All analyses controlled for household income, age, education level, race/ethnicity, rurality, gender identity, and job disruptions during the pandemic. We also included a dummy variable in all analyses to control for survey year (2021 or 2022). All analyses used survey weights to correct for income distribution because our sample overrepresents low-income households. Observations with missing data for any variables were dropped from the analyses. We report statistical significance as *p* ≤ 0.05. All analyses were done in Stata v17.0 [39].

## 3. Results

Table 2 presents sample characteristics. There were no major differences in outcomes by state; thus, Maine and Vermont respondents were combined for this analysis. The sample reflects the adult populations of Maine and Vermont with respect to race and ethnicity, education, and rurality. The sample overrepresents women and is slightly younger than the general population. The sample overrepresents low-income households, which we correct for in the models using survey weighting to maintain representativeness on income.

**Figure 1:**
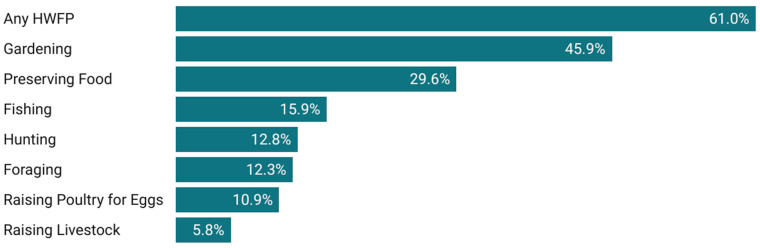
Share of respondents that indicated they engaged in HWFP activities in the last 12 months.

Among all respondents, 61.0% indicated that their household had engaged in an HWFP activity within 12 months of completing the survey. The most common activity was gardening (45.9%), followed by preserving food (29.6%), and fishing (15.9%) (Figure 1). The least common activities were raising livestock for meat or milk and raising poultry for eggs. Among respondents who engaged in HWFP, 55.5% reported engaging in two or more HWFP activities. For example, most (79.4%) foraging households also gardened and 61.4% of hunting households also fished.

### 3.1. HWFP Engagement and Food Security Status

Among the respondents who completed the food security module (*n* = 1890), 34.9% were food insecure at some point in the 12 months prior to taking the survey while 65.1% were food secure.

We used separate logistic regression models to examine the relationships between food security status and engaging in each individual HWFP activity or engaging in any HWFP (H1). We found that households that engaged in foraging (OR = 1.61, *p* = 0.006), hunting (OR = 1.64, *p* = 0.003), fishing (OR = 1.60, *p* = 0.005), raising livestock (OR = 2.71, *p* < 0.001), and raising poultry for eggs (OR = 2.07, *p* < 0.001) were more likely to be food insecure than households that did not do these activities (Table 3; full results of each model in Supplementary Materials, Tables S1–S8). Combining all HWFP activities together in a single model, we find no statistically significant relationship between HWFP engagement and food security status except for those that raised poultry for eggs (OR = 1.56; *p* = 0.041), who were significantly more likely to be food insecure (Table 4).

### 3.2. HWFP Engagement and Fruit and Vegetable Intake

Respondents reported an average fruit intake of 0.85 cup-eq/day and an average vegetable intake of 1.47 cup-eq/day, for a combined fruit and vegetable intake of 2.32 cup-eq/day. The 2020–2025 Dietary Guidelines for Americans (DGA) recommend that most adults consume 2 cup-eq of fruit per day and 2.5 to 3.5 cup-eq of vegetables per day [40]. Fewer than 2% of the respondents in our sample met these DGA recommendations for either fruit intake or vegetable intake.

When looking at fruit and vegetable intake in relation to engagement in relevant HWFP activities (H2a), we found that gardening, foraging, and preserving food were significantly positively associated with fruit, vegetable, and combined fruit and vegetable intake in all instances except fruit intake and foraging, for which no relationship was identified (Table 5; full results of each model in Supplementary Materials, Tables S9–S17). Specifically, respondents who gardened had a 0.09 cup-eq/day greater intake for vegetables (*p* < 0.001), 0.07 cup-eq/day for fruits (*p* < 0.001), and 0.15 cup-eq/day for combined fruit and vegetable intake (*p* < 0.001). These daily amounts equate to an additional 1.1 cup-eq of combined fruit and vegetable intake per week. Similarly, preserving food was associated with a 0.05 cup-eq/day greater intake of both vegetables and fruits (*p* = 0.028). Foraging was significantly associated with a 0.08 cup-eq/day greater intake for vegetables (*p* = 0.012) and a 0.11 cup-eq/day greater intake for fruits and vegetables combined (*p* = 0.038), more than an additional ¾ cup-eq over the course of one week. Exploring the relationship of these activities together in a single model demonstrated that collectively only gardening is associated with greater fruit and vegetable intake (0.14 cup-eq/day increase for fruits and vegetables combined (*p* < 0.001)) (Table 6; full results of each model in Supplementary Materials, Tables S18–S20).

We also explored the relationship between HWFP engagement and combined fruit and vegetable intake by food security status (H2b) (Table 7; full results of each model in Supplementary Materials, Tables S21–S24). Engaging in any HWFP activity is associated with greater combined fruit and vegetable intake among food insecure households (b = 0.14; *p* = 0.005) but not food secure households (*p* = 0.074). Participating in gardening is associated with greater combined fruit and vegetable intake among both food secure (b= 0.12; *p* = 0.006) and food insecure households (b = 0.22; *p* < 0.000). For food insecure households, this is the equivalent of nearly ¼ cup greater fruit and vegetable intake per day, or more than a cup and a half per week. Foraging (b = 0.18; *p* = 0.009) and food preservation (b = 0.10, *p* = 0.050) are also both associated with greater combined fruit and vegetable intake but only for food secure households. Looking at the combined effect of all three relevant HWFP activities in a single model, only gardening among food insecure households had a statistically significant effect on combined fruit and vegetable intake (Table 8). Among food insecure households, gardening was associated with a 0.23 cup-eq/day greater fruit and vegetable intake (*p* < 0.000), more than a cup and a half per week.

### 3.3. Hunting and Meat Consumption

**Figure 2:**
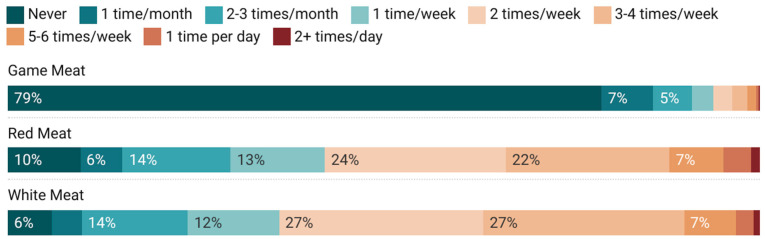
Consumption frequency of game meat, red meat, and white meat in the last 30 days.

Only 21.0% of respondents indicated they ate any game meat in the last 30 days, though 90.4% of respondents ate red meat and 94.1% ate white meat (Figure 2). Most (71.9%) respondents from households that hunted consumed game meat, while only 13.5% of respondents from households that did not hunt consumed game meat. Sixteen percent of food secure respondents consumed game meat, and 27.9% of food insecure respondents consumed game meat.

Respondents whose households engaged in hunting were significantly more likely to consume game meat than respondents whose households did not hunt (H3a) (OR = 17.25; *p* < 0.001) (Table 9; full results of each model in Supplementary Materials, Tables S25–S27). However, we also find that respondents who hunted were more likely to eat both red meat (OR = 1.91; *p* < 0.000) and white meat (OR = 1.87; *p* < 0.000) at greater frequencies than respondents who did not hunt (H3b). When looking at this relationship by food security status (H3c), both food secure (OR = 23.72, *p* < 0.001) and food insecure households that hunted (OR = 13.27, *p* < 0.001) were more likely to eat game meat than households that did not hunt (Table 10; full results of each model in Supplementary Materials, Tables S28–S30). Likewise, both food secure (OR = 1.99, *p* < 0.001) and food insecure households that hunted (OR = 2.23, *p* < 0.001) were more likely to consume red meat in greater frequencies than households that did not hunt. Only food insecure households that hunted OR = 1.75, *p* = 0.006) were more likely to consume white meat in greater frequencies than food insecure households that did not hunt.

## 4. Discussion

This study, using a large-scale representative population sample from two rural U.S. states examined the relationship of a suite of HWFP activities to food security and dietary intake. It provides new evidence, with a large population size, of the relationship between HWFP engagement and food security status, fruit, vegetable, and meat intake, with notable implications for both individual and population-level health, as well as for HWFP as a strategy for affecting these outcomes.

Overall, our evidence suggests that several HWFP activities, including foraging, hunting, fishing, raising livestock, and raising poultry for eggs, were associated with greater odds of being food insecure, consistent with some of our previous findings [10]. However, when combining all activities together into a single model, only households engaging in egg production were associated with greater odds of being food insecure. There was no statistically significant association between food security status and gardening or preserving food. The cross-sectional design of this study does not allow us to determine whether food security status influenced HWFP activity choice or vice versa. However, our results show that food insecure households were more likely to engage in certain activities than food secure households, perhaps as a coping strategy to supplement food from other sources. Notably, these activities have varying start-up costs, time commitment, required knowledge and skills, and type of food procured, which may influence who chooses to participate in each activity. For example, food insecure individuals may be more likely to hunt, fish, and raise livestock or poultry because animal products are relatively expensive foods to purchase at a store. 

In examining HWFP activities and their relationship to fruit and vegetable intake, we found that gardening, foraging, and preserving food are all positively and significantly associated with greater fruit and vegetable consumption. However, when combining all activities together into a single model, only gardening showed a significant association with greater fruit and vegetable consumption, with an approximately one cup-eq per week increase in consumption. Other studies have found that gardeners eat fruits and vegetables up to 2 more times per day than non-gardeners [17,18,19]. One possible reason that we found a smaller difference in fruit and vegetable intake between respondents from gardening and non-gardening households, compared to what previous research has found, is that our survey’s recall period was just 30 days, and it was conducted in the springtime, before gardeners in Vermont and Maine were harvesting significant quantities of anything other than asparagus, rhubarb, and tender greens. Gardeners consistently self-report that the seasons when they harvest from their gardens coincide with eating greater quantities of vegetables [14,16,41]. Given that the survey was conducted outside of harvest season, it is perhaps remarkable that we found a significant difference at all between gardeners’ and non-gardeners’ fruit and vegetable intakes and could be reflective of the high percentage of gardeners who also did food preservation or different food preferences between those who do and do not garden.

Furthermore, when exploring these relationships disaggregated by food security status, we find that this effect is stronger for food insecure households, especially in the combined model. As such, food insecure households engaging in gardening ate more than one and a half cup-eqs more fruit and vegetables per week, as compared to those not gardening. Food insecure households consume fewer fruits and vegetables overall compared to food secure households [42,43,44]. Furthermore, evidence early in the pandemic suggested that food insecure households were also more likely to reduce their fruit and vegetable intake [45,46]. Food insecure individuals are also more likely to have a suite of other diet-related health challenges such as hypertension, diabetes, and cardiovascular disease [47,48], and increased fruit and vegetable intake may reduce disease risk or prevalence [49].

Our analysis also looked more closely at the relationship between hunting and wild game meat consumption, a relationship that has been seldom, if ever, explored in population-level studies. We find an overall highly significant relationship between hunting and wild game consumption, which is especially true for food insecure respondents. Wild game meat can be an important source of protein and micronutrients and is typically lower in total and saturated fat than other sources of meat; thus, consuming game meat could have nutrition and health benefits [50]. However, in addition to being more likely to eat wild game meat, we also found that people who lived in households that engaged in hunting ate both red and white meat more frequently than people in non-hunting households. This suggests that hunting is not necessarily replacing other meat sources, but rather that it is being consumed in addition to red and white meat sources. This finding is consistent with previous research that showed people with connections to hunting or raising livestock reported more positive meat-related attitudes and more frequent meat consumption [51]. Future research should quantify the intake of different types of meat to examine whether people who hunt consume greater amounts of meat overall than people who do not hunt.

Overall, this study offers one of the largest explorations of HWFP in a high-income country and its relationship to food security and dietary intake of fruits, vegetables, and wild game. Strengths of the study are the large sample size, representativeness, and inclusion of multiple HWFP activities. The majority of households that engaged in HWFP did multiple types of HWFP rather than just one activity, which highlights the importance of looking at a suite of activities. Several limitations should be noted, which we suggest can be the focus of future research. First, our findings only show correlations between HWFP activities and dietary outcomes. We cannot establish causal relationships or control for other potential confounding factors that influence HWFP engagement and dietary outcomes, such as general health consciousness, available leisure time, or access to land and other resources needed to produce food. As noted, it may be that people who eat more fruits and vegetables or have a greater interest in healthy eating are more likely to garden, rather than the other way around. In reality, causation probably works in both directions to some extent, and only experimental research designs like that of Alaimo et al. (2023) [19] can begin to disentangle gardening’s effect on fruit and vegetable consumption from fruit and vegetable consumption’s effect on gardening. Even in that case, truly randomized control trials are not possible with HWFP, since gardening, hunting, fishing, and foraging are not interventions that can be randomly applied across a population. It would be unethical to keep people who want to do these activities from engaging in them, and it is not plausible to force people who are not interested in these activities to do them—and nearly half of respondents who did not engage in HWFP reported that they are not interested [52]. Even so, designing research that uses HWFP activities as an intervention may be a worthwhile direction for future research that can generate policy-relevant findings about the potential nutrition, health, and food sovereignty benefits of food self-provisioning. Furthermore, longitudinal studies are needed to determine causality.

Another limitation of this study is the dietary assessment method. The DSQ and the associated scoring algorithms only provide estimated intakes of fruits and vegetables based on respondent reports of how frequently they eat those foods. Additionally, there is no way to estimate intake of meat so our findings are limited to examining meat intake frequency rather than quantity. Other dietary assessment methods, such as food records or a series of 24-h recalls could be used to more precisely measure intake of specific food groups in future research. Furthermore, there was a mismatch between the period about which we asked about household HWFP engagement (the last year) and individual diet (the last 30 days). Furthermore, there is a seasonal mismatch: the 30-day diet recall period did not fall during the half of the year when gardens are actively producing. The fact that members of gardening households still report eating more fruits and vegetables than non-gardeners could suggest early-season harvests or preserved bounty from the previous year, but it could also suggest that causation is in fact reversed, and households take up gardening because they eat more fruits and vegetables.

Finally, this studied assessed HWFP engagement using a simple binary measurement of participation. This does not capture the variation in intensity of these activities, which could significantly influence the effects of participation on food security and dietary intake. Furthermore, participants likely have varying levels of success in procuring food through these activities and some respondents may fail to procure any food. For example, someone who hunts may or may not successfully harvest an animal in a given year. Likewise, someone who hunts frequently or hunts several species across the year may procure more food than someone who only hunts once a year or who only hunts for a single species. Future research should measure participation at a more granular level and attempt to quantify the amount of food procured through these activities to more clearly understand the relationships between HWFP participation, food security, and dietary intake.

This study provides new evidence of the relationships between HWFP engagement and food security status and dietary intake in a high-income country. The results may provide insights for other high-income settings, though results would likely vary by region due to differences in rurality, climate and growing seasons, access to land, cultural traditions and norms around hunting and fishing, and regulations around keeping livestock. This study focuses on Northern New England, which is a largely rural area and has a strong cultural and historical tradition of food self-provisioning, especially through hunting. Furthermore, the generalizability of our results may be affected by differences between the survey sample and study population for some target demographics and any non-response bias resulting from the recruitment process and sampling strategy. The sample overrepresents women and respondents were slightly younger than the population. Gender and age may influence an individual’s likeliness of engaging in HWFP activities, experiencing food security, and dietary choices. For example, women may be less likely to hunt or fish than men, and therefore, our sample may underestimate the number of people who engage in these activities.

## 5. Conclusions

In this study, we show that HWFP engagement is associated with food security status. Respondents who engaged in these activities were more likely to be food insecure, indicating that food insecure households may be using HWFP as a coping strategy. We also find a positive association between HWFP engagement and fruit and vegetable intake, which may have important health implications, especially for food insecure individuals who typically have lower fruit and vegetable intakes than food secure individuals. Finally, we show a positive association between hunting and consumption of wild game meat. Our findings add to emerging evidence on the public health and food security benefits of HWFP engagement. We suggest that future research continues to examine these relationships over time to assess whether HWFP is an effective long-term strategy to improve health, food security, and food sovereignty.

## Figures and Tables

**Table 1:** Complete list of variables included in the analysis.

Variable Name	Question(s)	Scale
Food Security Status	USDA 6-item food security model for past 12 months	0 = Food Secure; 1 = Food Insecure
**Dietary Quality Variables**		
Fruit and Vegetable Intake	Predicted intake of fruits and vegetables including legumes and excluding French fries based on DSQ	Original DSQ scale: 1 = Never; 2 = 1 time in the last month; 3 = 2–3 times in the last month; 4 = 1 time per week; 5 = 2 times per week; 6 = 3–4 times per week; 7 = 5–6 times per week; 8 = 1 time per day; 9 = 2 or more times per day
	
Fruit Intake	Predicted intake of fruits (including 100% pure fruit juice) based on DSQ	
Vegetable Intake	Predicted intake of vegetables excluding French fries based on DSQ	Estimated intake reported in Cup Equivalents Per Day calculated using the scoring algorithms developed by the National Cancer Institute.
	
Game Meat Consumption	During the past month, how often did you eat wild game meat such as venison, wild turkey, pheasant, or bear?	0 = Did Not Consume; 1 = Did Consume
		
Red Meat Consumption	During the past month, how often did you eat red meat, such as beef, pork, ham, or sausage?	1 = Never; 2 = 1 time in the last month; 3 = 2–3 times in the last month; 4 = 1 time per week; 5 = 2 times per week; 6 = 3–4 times per week; 7 = 5–6 times per week; 8 = 1 time per day; 9 = 2 or more times per day
	
White Meat Consumption	During the past month, how often did you eat white meat, such as chicken and turkey?	
**Home and Wild Food Procurement (HWFP) Variables**		
Gardening	Has your household engaged in any of these activities in the last 12 months?	0 = No; 1 = Yes
Foraging		
Fishing		
Hunting		
Raising Livestock		
Raising Poultry for Eggs		
Preserving Food		
		
Any HWFP	Variable created based on respondent indicating that they engaged in any of the individual HWFP activities	0 = No; 1 = Yes
**Demographic Variables**		
Annual Household Income	Which of the following best describes your household income range before taxes? [2021 survey asked about 2019 income; 2022 survey asked about 2021 income]	0 = Household income < $50,000 1 = Household income ≥ $50,000
		
College Degree	What is the highest level of formal education that you have completed?	0 = Less than associate’s degree 1 = Associate’s or higher
Age	In what year were you born?	0 = Under 65; 1 = 65 and older

Race	What is your race? Check all that apply.	Response options include: American Indian/Alaska Native; Asian Indian; Black or African American; Chamorro; Chinese; Filipino; Japanese; Korean; Native Hawaiian; Samoan; Vietnamese; White
		
Ethnicity	Are you of Hispanic, Latino, or Spanish origin?	1 = No, not Hispanic, Latino, or Spanish origin; 2 = Yes, Mexican, Mexican American, Chicano; 3 = Yes, Puerto Rican; 4 = Yes, Cuban; 5 = Yes, Hispanic, Latino, or Spanish origin
		
Race/Ethnicity Binary	Based on responses to race and ethnicity questions above	0 = BIPOC; 1 = non-Hispanic White
		
Gender Identity	Which of the following best describes your gender identity?	0 = Male; 1 = Female; 2 = Another Gender Identity
		
Rural/urban classification	Rural or urban classification based on zip code responses and RUCA codes	0 = Rural; 1 = Urban
		
Job Disruption	Have you or anyone in your household experienced a loss of income, reduction of hours, furlough or job loss since the COVID-19 outbreak began 11 March 2020?	0 = No; 1 = Yes

**Table 2:** Demographic characteristics of survey respondents (*n* = 2001).

Category	Characteristic	Sample		Population ^1^
		n	%	%
Age	18–29	360	18.0	18.1
	30–44	578	28.9	21.7
	45–64	638	31.9	35.3
	≥65	425	21.2	25.0
				
Gender	Male	657	32.8	49.4
	Female	1308	65.4	50.6
	Other Gender	28	1.4	n/a
				
Race/Ethnicity	Non-Hispanic White	1827	91.3	93.1
	BIPOC	157	7.9	6.9
				
Education Level	No College Degree	1052	52.6	54.4
	College Degree	949	47.4	45.6
				
Household Income	<USD50,000/year	973	48.6	38.8
	≥USD50,000/year	1027	51.3	61.2
				
Rurality	Urban	853	42.7	46.5
	Rural	1143	57.1	53.5
				
Job Disruption	Yes	1230	61.5	n/a
	No	770	38.5	n/a

^1^ Based on US Census Bureau 2017–2021 American Community Survey estimates and 2010 Decennial Census data for Maine and Vermont. Categories do not sum to 100% due to missing data.

**Table 3:** Summary of results from separate logistic regression models predicting the odds of food insecurity by overall and specific HWFP activity engagement.

Activity	Odds Ratio	Standard Error	*p*-Value	95% CI		Accuracy Rate (%) ^1^
Any HWFP	1.19	0.141	0.132	0.948	1.504	73.3
Gardening	1.06	0.123	0.606	0.846	1.331	73.2
Preserving Food	1.22	0.159	0.128	0.945	1.574	73.3
**Foraging**	**1.61**	**0.275**	**0.006**	**1.149**	**2.248**	73.7
**Hunting**	**1.64**	**0.277**	**0.003**	**1.177**	**2.283**	73.4
**Fishing**	**1.60**	**0.264**	**0.005**	**1.156**	**2.208**	73.2
**Raising Livestock**	**2.71**	**0.733**	**0.000**	**1.592**	**4.602**	73.9
**Raising Poultry for Eggs**	**2.07**	**0.390**	**0.000**	**1.433**	**2.997**	73.9

Odds ratios higher than 1.00 indicate a greater odds of food insecurity. Each model also included control variables for age, gender identity, race/ethnicity, education level, income, rurality, job disruption, and survey year. Statistically significant (*p* < 0.05) results are bolded for emphasis. ^1^ Ratio of correctly predicted observations to the total number of observations.

**Table 4:** Results of logistic regression model predicting the combined effects of HWFP engagement on food security status (accuracy rate ^1^ = 74.5%).

Variable	Odds Ratio	Standard Error	*p*-Value	95% CI	
Gardening	0.85	0.110	0.204	0.656	1.094
Preserving Food	0.99	0.148	0.987	0.745	1.335
Foraging	1.27	0.239	0.197	0.882	1.839
Hunting	1.08	0.217	0.716	0.725	1.599
Fishing	1.24	0.242	0.272	0.845	1.818
Raising Livestock	1.71	0.535	0.089	0.922	3.154
**Raising Poultry for Eggs**	**1.56**	**0.339**	**0.041**	**1.018**	**2.390**
Race/Ethnicity	0.75	0.154	0.156	0.497	1.118
Gender Identity	1.08	0.136	0.536	0.845	1.383
**Age**	**0.32**	**0.053**	**0.000**	**0.228**	**0.438**
**Education Level**	**0.52**	**0.063**	**0.000**	**0.408**	**0.656**
**Household Income**	**0.25**	**0.031**	**0.000**	**0.199**	**0.322**
Rurality	1.05	0.121	0.670	0.839	1.315
**Job Disruption**	**3.54**	**0.417**	**0.000**	**2.814**	**4.462**
**Survey Year**	**1.64**	**0.197**	**0.000**	**1.297**	**2.076**

Odds ratios higher than 1.00 indicate a greater odds of food insecurity. Statistically significant (*p* < 0.05) results are bolded for emphasis. ^1^ Ratio of correctly predicted observations to the total number of observations.

**Table 5:** Summary of results from separate linear regression models predicting the effects of HWFP engagement on daily fruit and vegetable intake (cup equivalents).

HWFP Activity	Diet Factor	Coefficient	Standard Error	*p*-Value	95% CI	
Gardening	**Vegetable Intake**	**0.09**	**0.020**	**0.000**	**0.049**	**0.130**
	**Fruit Intake**	**0.07**	**0.019**	**0.000**	**0.031**	**0.104**
	**Fruit and Vegetable Intake**	**0.15**	**0.033**	**0.000**	**0.087**	**0.217**
Preserving Food	**Vegetable Intake**	**0.05**	**0.022**	**0.028**	**0.005**	**0.093**
	**Fruit Intake**	**0.05**	**0.021**	**0.018**	**0.009**	**0.093**
	**Fruit and Vegetable Intake**	**0.09**	**0.037**	**0.019**	**0.014**	**0.160**
Foraging	**Vegetable Intake**	**0.08**	**0.030**	**0.012**	**0.017**	**0.138**
	Fruit Intake	0.03	0.028	0.248	−0.023	0.089
	**Fruit and Vegetable Intake**	**0.11**	**0.051**	**0.038**	**0.005**	**0.210**

Each model also included control variables for age, gender identity, race/ethnicity, education level, income, rurality, job disruption, and survey year. Statistically significant (*p* < 0.05) results are bolded for emphasis.

**Table 6:** Summary of results for linear regression models predicting the combined effects of HWFP engagement on daily fruit and vegetable intake (cup equivalents).

Diet Factor	HWFP Activity	Coefficient	Standard Error	*p*-Value	95% CI	
Vegetable Intake	**Gardening**	**0.08**	**0.022**	**0.000**	**0.034**	**0.120**
	Foraging	0.04	0.034	0.190	−0.022	0.059
	Preserving Food	0.01	0.024	0.627	−0.035	0.059
Fruit Intake	**Gardening**	**0.06**	**0.021**	**0.005**	**0.018**	**0.099**
	Foraging	0.00	0.030	0.981	−0.059	0.060
	Preserving Food	0.03	0.023	0.212	−0.016	0.074
Fruit and Vegetable Intake	**Gardening**	**0.14**	**0.037**	**0.000**	**0.064**	**0.207**
	Foraging	0.04	0.056	0.421	−0.064	0.154
	Preserving Food	0.03	0.040	0.487	−0.050	0.207

Each model also included control variables for age, gender identity, race/ethnicity, education level, income, rurality, job disruption, and survey year. Statistically significant (*p* < 0.05) results are bolded for emphasis.

**Table 7:** Summary of results from separate linear regression models predicting the effects of HWFP engagement on daily combined fruit and vegetable intake (cup equivalents), by food security status.

HWFP Activity	Food Security Status	Coefficient	Standard Error	*p*-Value	95% CI	
Any HWFP	Food Secure	0.08	0.043	0.074	−0.017	0.150
	**Food Insecure**	**0.14**	**0.050**	**0.005**	**0.113**	**0.327**
Gardening	**Food Secure**	**0.12**	**0.043**	**0.006**	**0.035**	**0.200**
	**Food Insecure**	**0.22**	**0.054**	**0.000**	**0.100**	**0.327**
Preservation	**Food Secure**	**0.10**	**0.048**	**0.050**	**0.000**	**0.192**
	Food Insecure	0.07	0.060	0.245	−0.048	0.188
Foraging	**Food Secure**	**0.18**	**0.069**	**0.009**	**0.045**	**0.320**
	Food Insecure	0.02	0.079	0.851	−0.142	0.172

Each model also included control variables for age, gender identity, race/ethnicity, education level, income, rurality, job disruption, and survey year. Statistically significant (*p* < 0.05) results are bolded for emphasis.

**Table 8:** Linear regression results predicting the combined effects of gardening, food preservation, and foraging engagement on daily fruit and vegetable intake (cup equivalents), by food security status.

Fruit and Vegetable Intake	Coefficient	Standard Error	*p*-Value	95% CI	
Food Secure					
Gardening	0.087	0.047	0.065	−0.006	0.179
Preserving Food	0.043	0.052	0.412	−0.059	0.145
Foraging	0.139	0.076	0.069	−0.011	0.288
Race/Ethnicity	−0.115	0.083	0.166	−0.278	0.048
**Gender Identity**	**−0.213**	**0.046**	**0.000**	**−0.304**	**−0.123**
Age	0.049	0.048	0.303	−0.045	0.143
Education Level	0.057	0.045	0.206	−0.031	0.145
**Household Income**	**0.176**	**0.043**	**0.000**	**0.091**	**0.261**
Rurality	−0.053	0.043	0.220	−0.138	0.032
Job Disruption	0.066	0.048	0.165	−0.027	0.160
**Survey Year**	**0.113**	**0.044**	**0.010**	**0.027**	**0.199**
Food Insecure					
**Gardening**	**0.235**	**0.062**	**0.000**	**0.113**	**0.357**
Preserving Food	0.009	0.061	0.882	−0.111	0.129
Foraging	−0.085	0.084	0.310	−0.250	0.080
Race/Ethnicity	−0.047	0.075	0.535	−0.194	0.101
**Gender Identity**	**−0.347**	**0.064**	**0.000**	**−0.473**	**−0.221**
Age	0.013	0.085	0.875	−0.154	0.180
Education Level	0.056	0.061	0.359	−0.064	0.177
Household Income	0.078	0.058	0.178	−0.036	0.191
Rurality	0.095	0.056	0.092	−0.015	0.204
Job Disruption	−0.014	0.053	0.785	−0.118	0.089
Survey Year	−0.101	0.056	0.072	−0.212	0.009

Statistically significant (*p* < 0.05) results are bolded for emphasis.

**Table 9:** Summary of results from separate logistic and ordinal logistic regression models predicting the effects of hunting on frequency of meat intake.

Dietary Factor	Odds Ratio	Standard Error	*p*-Value	95% CI		Accuracy Rate ^1^ (%)
**Game Meat**	**17.25**	**3.050**	**0.000**	**12.193**	**24.390**	84.1
**Red Meat**	**1.91**	**0.227**	**0.000**	**1.513**	**2.411**	n/a
**White Meat**	**1.87**	**0.192**	**0.000**	**1.527**	**2.287**	n/a

Each model also included control variables for age, gender identity, race/ethnicity, education level, income, rurality, job disruption, and survey year. Statistically significant (*p* < 0.05) results are bolded for emphasis. ^1^ Ratio of correctly predicted observations to the total number of observations.

**Table 10:** Summary of results from separate logistic and ordinal logistic regression models predicting the effects of hunting on frequency of meat intake, by food security status.

Dietary Factor	Food Security Status	Odds Ratio	Standard Error	*p*-Value	95% CI		Accuracy Rate ^1^ (%)
Game Meat	**Food Secure**	**23.72**	**5.854**	**0.000**	**14.621**	**38.474**	68.9
	**Food Insecure**	**13.27**	**3.656**	**0.000**	**7.731**	**22.767**	68.9
Red Meat	**Food Secure**	**1.99**	**0.327**	**0.000**	**1.446**	**2.749**	n/a
	**Food Insecure**	**2.23**	**0.435**	**0.000**	**1.520**	**3.267**	n/a
White Meat	Food Secure	1.25	0.183	0.134	0.935	1.660	n/a
	**Food Insecure**	**1.75**	**0.354**	**0.006**	**1.177**	**2.602**	n/a

The model also included control variables for age, gender identity, race/ethnicity, education level, income, rurality, job disruption, and survey year. Statistically significant (*p* < 0.05) results are bolded for emphasis. ^1^ Ratio of correctly predicted observations to the total number of observations.

## Data Availability

The datasets used and analyzed during the current study are available from the corresponding author on reasonable request due to privacy considerations.

## Supplementary Materials

The following supporting information can be downloaded at: https://www.mdpi.com/article/10.3390/nu17101627/s1.

## Abbreviations

The following abbreviations are used in this manuscript:

HWFP	Home and wild food procurement
DSQ	Dietary Screener Questionnaire
DGA	Dietary Guidelines for Americans
