# Supplementary material for: Home and Wild Food Procurement Were Associated with Greater Intake of Fruits and Vegetables During the COVID-19 Pandemic in Northern New England in a Cross-Sectional Study
Source: Nutrients. 2025 May 9;17(10):1627. doi: 10.3390/nu17101627 (PMC12114189; doi:10.3390/nu17101627)
Supplement: Supplementary file 1 [file nutrients-17-01627-s001.zip › nutrients-3611537-supplementary.pdf]

Supplementary Materials

Table S1. Complete list of variables included in the analysis

Variable Name	Question(s)	Scale
Food Security Status	USDA 6-item food security model for past 12 months	0 = Food Secure; 1 = Food Insecure
	**Dietary Quality Variables**	
Fruit and Vegetable Intake	Predicted intake of fruits and vegetables including legumes and excluding French fries based on DSQ	Cup Equivalents Per Day
Fruit Intake	Predicted intake of fruits (including 100% pure fruit juice) based on DSQ	Cup Equivalents Per Day
Vegetable Intake	Predicted intake of vegetables excluding French fries based on DSQ	Cup Equivalents Per Day
Game Meat Consumption	During the past month, how often did you eat wild game meat such as venison, wild turkey, pheasant, or bear?	0 = Did Not Consume; 1 = Did Consume
Red Meat Consumption	During the past month, how often did you eat red meat, such as beef, pork, ham, or sausage?	1 = Never; 2 = 1 time in the last month; 3 = 2-3 times in the last month; 4 = 1 time per week; 5 = 2 times per week; 6 = 3-4 times per week; 7 = 5-6 times per week; 8 = 1 time per day; 9 = 2 or more times per day
White Meat Consumption	During the past month, how often did you eat white meat, such as chicken and turkey?	
	**Home and Wild Food Procurement (HWFP) Variables**	
Gardening<br>Foraging<br>Fishing<br>Hunting<br>Raising Livestock<br>Raising Poultry for Eggs<br>Preserving Food	Has your household engaged in any of these activities in the following in the last 12 months?	0 = No; 1 = Yes

Any HWFP	Variable created based on respondent indicating that they engaged in any of the individual HWFP activities	0 = No; 1 = Yes
**Demographic Variables**		
Annual Household Income	Which of the following best describes your household income range before taxes? [2021 survey asked about 2019 income; 2022 survey asked about 2021 income]	0 = Household income < $50,000<br>1 = Household income ≥ $50,000
College Degree	What is the highest level of formal education that you have completed?	0 = Less than associate's degree<br>1 = Associate's or higher
Age	In what year were you born?	0 = Under 65; 1 = 65 and older
Race	What is your race? Check all that apply.	Response options include: American Indian/Alaska Native; Asian Indian; Black or African American; Chamorro; Chinese; Filipino; Japanese; Korean; Native Hawaiian; Samoan; Vietnamese; White
Ethnicity	Are you of Hispanic, Latino, or Spanish origin?	1 = No, not Hispanic, Latino, or Spanish origin; 2 = Yes, Mexican, Mexican American, Chicano; 3 = Yes, Puerto Rican; 4 = Yes, Cuban; 5 = Yes, Hispanic, Latino, or Spanish origin
Race/Ethnicity Binary	Based on responses to race and ethnicity questions above	0 = BIPOC; 1 = non-Hispanic White
Gender Identity	Which of the following best describes your gender identity?	0 = Male; 1 = Female; 3 = Another Gender Identity
Rural/urban classification	Rural or urban classification based on zip code responses and RUCA codes	0 = Rural; 1 = Urban

Job Disruption	Have you or anyone in your household experienced a loss of income, reduction of hours, furlough or job loss since the COVID-19 outbreak began March 11th, 2020?	0 = No; 1 = Yes

Table S2. Logistic regression results predicting the odds of food insecurity by any HWFP activity engagement. Odds ratios higher than 1.00 indicate a greater odds of food insecurity. Statistically significant (p<0.05) results are bolded for emphasis.

	**Odds Ratio**	**Standard Error**	**p-value**	**95% CI**	
Any HWFP	1.19	0.141	0.132	0.948	1.504
Race/Ethnicity	0.75	0.156	0.165	0.498	1.126
Gender Identity	1.00	0.124	0.996	0.784	1.277
**Age**	**0.29**	**0.048**	**0.000**	**0.210**	**0.401**
**Education Level**	**0.51**	**0.061**	**0.000**	**0.400**	**0.640**
**Household Income**	**0.25**	**0.030**	**0.000**	**0.199**	**0.319**
Rurality	1.02	0.117	0.845	0.817	1.280
**Job Disruption**	**3.53**	**0.410**	**0.000**	**2.808**	**4.428**
**Survey Year**	**1.54**	**0.179**	**0.000**	**1.226**	**1.932**

Table S3. Logistic regression results predicting the odds of food insecurity by gardening. Odds ratios higher than 1.00 indicate a greater odds of food insecurity. Statistically significant (p<0.05) results are bolded for emphasis.

	**Odds Ratio**	**Standard Error**	**p-value**	**95% CI**	
Gardening	1.06	0.123	0.606	0.846	1.331
Race/Ethnicity	0.75	0.157	0.173	0.501	1.133
Gender Identity	0.99	0.124	0.963	0.779	1.269
**Age**	**0.29**	**0.048**	**0.000**	**0.209**	**0.400**
**Education Level**	**0.51**	**0.061**	**0.000**	**0.403**	**0.645**
**Household Income**	**0.26**	**0.031**	**0.000**	**0.201**	**0.323**
Rurality	1.02	0.116	0.887	0.812	1.271
**Job Disruption**	**3.54**	**0.411**	**0.000**	**2.823**	**4.449**
**Survey Year**	**1.56**	**0.180**	**0.000**	**1.240**	**1.951**

Table S4. Logistic regression results predicting the odds of food insecurity by foraging. Odds ratios higher than 1.00 indicate a greater odds of food insecurity. Statistically significant (p<0.05) results are bolded for emphasis.

	Odds Ratio	Standard Error	p-value	95% CI	
**Foraging**	**1.61**	**0.275**	**0.006**	**1.149**	**2.248**
Race/Ethnicity	0.75	0.158	0.174	0.498	1.134
Gender Identity	1.04	0.131	0.750	0.813	1.332
**Age**	**0.29**	**0.049**	**0.000**	**0.212**	**0.406**
**Education Level**	**0.51**	**0.061**	**0.000**	**0.401**	**0.642**
**Household Income**	**0.25**	**0.031**	**0.000**	**0.199**	**0.319**
Rurality	1.04	0.120	0.730	0.831	1.303
**Job Disruption**	**3.48**	**0.406**	**0.000**	**2.773**	**4.378**
**Survey Year**	**1.58**	**0.183**	**0.000**	**1.259**	**1.983**

Table S5. Logistic regression results predicting the odds of food insecurity by hunting. Odds ratios higher than 1.00 indicate a greater odds of food insecurity. Statistically significant (p<0.05) results are bolded for emphasis.

	Odds Ratio	Standard Error	p-value	95% CI	
**Hunting**	**1.64**	**0.277**	**0.003**	**1.177**	**2.283**
Race/Ethnicity	0.75	0.156	0.170	0.501	1.130
Gender Identity	1.02	0.127	0.875	0.798	1.303
**Age**	**0.30**	**0.049**	**0.000**	**0.216**	**0.412**
**Education Level**	**0.51**	**0.061**	**0.000**	**0.401**	**0.641**
**Household Income**	**0.25**	**0.030**	**0.000**	**0.199**	**0.319**
Rurality	1.03	0.118	0.791	0.823	1.291
**Job Disruption**	**3.58**	**0.416**	**0.000**	**2.847**	**4.491**
**Survey Year**	**1.60**	**0.185**	**0.000**	**1.273**	**2.004**

Table S6. Logistic regression results predicting the odds of food insecurity by fishing. Odds ratios higher than 1.00 indicate a greater odds of food insecurity. Statistically significant (p<0.05) results are bolded for emphasis.

	**Odds Ratio**	**Standard Error**	**p-value**	**95% CI**	
**Fishing**	**1.60**	**0.264**	**0.005**	**1.156**	**2.208**
Race/Ethnicity	0.76	0.160	0.195	0.504	1.151
Gender Identity	1.04	0.129	0.765	0.813	1.325
**Age**	**0.30**	**0.049**	**0.000**	**0.218**	**0.414**
**Education Level**	**0.51**	**0.061**	**0.000**	**0.400**	**0.640**
**Household Income**	**0.25**	**0.031**	**0.000**	**0.200**	**0.320**
Rurality	1.04	0.120	0.710	0.833	1.307
**Job Disruption**	**3.53**	**0.411**	**0.000**	**2.809**	**4.436**
**Survey Year**	**1.56**	**0.180**	**0.000**	**1.240**	**1.952**

Table S7. Logistic regression results predicting the odds of food insecurity by preserving food. Odds ratios higher than 1.00 indicate a greater odds of food insecurity. Statistically significant (p<0.05) results are bolded for emphasis.

	**Odds Ratio**	**Standard Error**	**p-value**	**95% CI**	
Preserving Food	1.22	0.159	0.128	0.945	1.574
Race/Ethnicity	0.75	0.156	0.168	0.499	1.129
Gender Identity	0.99	0.124	0.954	0.778	1.267
**Age**	**0.29**	**0.047**	**0.000**	**0.209**	**0.398**
**Education Level**	**0.50**	**0.061**	**0.000**	**0.397**	**0.637**
**Household Income**	**0.26**	**0.031**	**0.000**	**0.202**	**0.323**
Rurality	1.01	0.116	0.908	0.809	1.269
**Job Disruption**	**3.52**	**0.409**	**0.000**	**2.798**	**4.417**
**Survey Year**	**1.53**	**0.179**	**0.000**	**1.213**	**1.920**

Table S8. Logistic regression results predicting the odds of food insecurity by raising livestock. Odds ratios higher than 1.00 indicate a greater odds of food insecurity. Statistically significant (p<0.05) results are bolded for emphasis.

	Odds Ratio	Standard Error	p-value	95% CI	
**Raising Livestock**	**2.71**	**0.733**	**0.000**	**1.592**	**4.602**
Race/Ethnicity	0.74	0.153	0.142	0.491	1.108
Gender Identity	1.04	0.130	0.751	0.815	1.328
**Age**	**0.30**	**0.050**	**0.000**	**0.218**	**0.417**
**Education Level**	**0.51**	**0.061**	**0.000**	**0.406**	**0.648**
**Household Income**	**0.26**	**0.031**	**0.000**	**0.202**	**0.325**
Rurality	1.02	0.117	0.856	0.816	1.278
**Job Disruption**	**3.60**	**0.419**	**0.000**	**2.865**	**4.523**
**Survey Year**	**1.65**	**0.192**	**0.000**	**1.310**	**2.069**

Table S9. Logistic regression results predicting the odds of food insecurity by raising poultry for eggs. Odds ratios higher than 1.00 indicate a greater odds of food insecurity. Statistically significant (p<0.05) results are bolded for emphasis.

	Odds Ratio	Standard Error	p-value	95% CI	
**Raising Poultry for Eggs**	**2.07**	**0.390**	**0.000**	**1.433**	**2.997**
Race/Ethnicity	0.75	0.154	0.163	0.502	1.123
Gender Identity	1.01	0.125	0.906	0.796	1.293
**Age**	**0.30**	**0.050**	**0.000**	**0.220**	**0.420**
**Education Level**	**0.52**	**0.062**	**0.000**	**0.409**	**0.654**
**Household Income**	**0.25**	**0.031**	**0.000**	**0.199**	**0.320**
Rurality	1.03	0.118	0.803	0.822	1.288
**Job Disruption**	**3.53**	**0.411**	**0.000**	**2.813**	**4.438**
**Survey Year**	**1.58**	**0.183**	**0.000**	**1.259**	**1.983**

Table S10. Linear regression results predicting the effects of gardening on daily fruit and vegetable intake (cup equivalents). Statistically significant (p<0.05) results are bolded for emphasis.

**Fruit and Vegetable Intake**	**Coefficient**	**Standard Error**	**p-value**	**95% CI**	
**Gardening**	**0.152**	**0.033**	**0.000**	**0.087**	**0.217**
Race/Ethnicity	-0.044	0.058	0.450	-0.157	0.070
**Gender Identity**	**-0.254**	**0.036**	**0.000**	**-0.325**	**-0.184**
**Age**	**0.085**	**0.039**	**0.030**	**0.008**	**0.162**
**Education Level**	**0.089**	**0.034**	**0.010**	**0.021**	**0.156**
**Household Income**	**0.194**	**0.034**	**0.000**	**0.128**	**0.260**
Rurality	0.009	0.034	0.779	-0.057	0.076
Job Disruption	-0.004	0.035	0.901	-0.073	0.064
Survey Year	0.033	0.032	0.304	-0.030	0.097

Table S11. Linear regression results predicting the effects of gardening on daily fruit intake (cup equivalents). Statistically significant (p<0.05) results are bolded for emphasis.

**Fruit Intake**	**Coefficient**	**Standard Error**	**p-value**	**95% CI**	
**Gardening**	**0.068**	**0.019**	**0.000**	**0.031**	**0.104**
Race/Ethnicity	-0.051	0.038	0.175	-0.124	0.023
**Gender Identity**	**-0.053**	**0.021**	**0.011**	**-0.094**	**-0.012**
Age	-0.002	0.021	0.919	-0.044	0.040
**Education Level**	**0.038**	**0.019**	**0.049**	**0.000**	**0.075**
**Household Income**	**0.055**	**0.019**	**0.004**	**0.018**	**0.092**
Rurality	0.002	0.019	0.907	-0.035	0.040
Job Disruption	-0.005	0.020	0.793	-0.044	0.034
Survey Year	0.019	0.018	0.305	-0.017	0.054

Table S12. Linear regression results predicting the effects of gardening on daily vegetable intake (cup equivalents). Statistically significant (p<0.05) results are bolded for emphasis.

Vegetable Intake	Coefficient	Standard Error	p-value	95% CI	
**Gardening**	**0.088**	**0.020**	**0.000**	**0.049**	**0.127**
Race/Ethnicity	-0.017	0.034	0.612	-0.084	0.049
**Gender Identity**	**-0.209**	**0.022**	**0.000**	**-0.252**	**-0.167**
**Age**	**0.082**	**0.025**	**0.001**	**0.033**	**0.131**
Education Level	0.036	0.021	0.085	-0.005	0.077
**Household Income**	**0.120**	**0.020**	**0.000**	**0.081**	**0.160**
Rurality	0.011	0.021	0.581	-0.029	0.052
Job Disruption	0.012	0.021	0.576	-0.030	0.053
Survey Year	0.016	0.020	0.410	-0.022	0.055

Table S13. Linear regression results predicting the effects of preserving food on daily fruit and vegetable intake (cup equivalents). Statistically significant (p<0.05) results are bolded for emphasis.

Fruit and Vegetable Intake	Coefficient	Standard Error	p-value	95% CI	
**Preservation**	**0.087**	**0.037**	**0.019**	**0.014**	**0.160**
Race/Ethnicity	-0.048	0.058	0.408	-0.162	0.066
**Gender Identity**	**-0.256**	**0.036**	**0.000**	**-0.328**	**-0.185**
**Age**	**0.091**	**0.040**	**0.022**	**0.013**	**0.169**
**Education Level**	**0.094**	**0.035**	**0.007**	**0.025**	**0.163**
**Household Income**	**0.207**	**0.034**	**0.000**	**0.142**	**0.273**
Rurality	0.002	0.034	0.962	-0.065	0.068
Job Disruption	-0.004	0.035	0.912	-0.073	0.066
Survey Year	0.029	0.033	0.379	-0.036	0.094

Table S14. Linear regression results predicting the effects of preserving food on daily fruit intake (cup equivalents). Statistically significant (p<0.05) results are bolded for emphasis.

Fruit Intake	Coefficient	Standard Error	p-value	95% CI	
**Preservation**	**0.051**	**0.021**	**0.018**	**0.009**	**0.093**
Race/Ethnicity	-0.053	0.038	0.158	-0.127	0.021
**Gender Identity**	**-0.054**	**0.021**	**0.011**	**-0.096**	**-0.012**
Age	0.000	0.021	0.993	-0.042	0.042
**Education Level**	**0.039**	**0.019**	**0.044**	**0.001**	**0.077**
**Household Income**	**0.061**	**0.019**	**0.001**	**0.024**	**0.098**
Rurality	-0.001	0.019	0.951	-0.039	0.036
Job Disruption	-0.006	0.020	0.767	-0.045	0.034
Survey Year	0.015	0.019	0.409	-0.021	0.052

Table S15. Linear regression results predicting the effects of preserving food on daily vegetable intake (cup equivalents). Statistically significant (p<0.05) results are bolded for emphasis.

Vegetable Intake	Coefficient	Standard Error	p-value	95% CI	
**Preservation**	**0.049**	**0.022**	**0.028**	**0.005**	**0.093**
Race/Ethnicity	-0.020	0.034	0.562	-0.086	0.047
**Gender Identity**	**-0.210**	**0.022**	**0.000**	**-0.253**	**-0.167**
**Age**	**0.085**	**0.025**	**0.001**	**0.036**	**0.134**
Education Level	0.039	0.021	0.065	-0.002	0.081
**Household Income**	**0.128**	**0.020**	**0.000**	**0.088**	**0.168**
Rurality	0.007	0.021	0.740	-0.033	0.047
Job Disruption	0.012	0.021	0.570	-0.030	0.054
Survey Year	0.014	0.020	0.485	-0.025	0.053

Table S16. Linear regression results predicting the effects of foraging on daily fruit and vegetable intake (cup equivalents). Statistically significant ($p<0.05$) results are bolded for emphasis.

**Fruit and Vegetable Intake**	**Coefficient**	**Standard Error**	**p-value**	**95% CI**	
**Foraging**	**0.106**	**0.051**	**0.038**	**0.006**	**0.206**
Race/Ethnicity	-0.045	0.058	0.444	-0.159	0.070
**Gender Identity**	**-0.247**	**0.037**	**0.000**	**-0.319**	**-0.174**
**Age**	**0.101**	**0.040**	**0.012**	**0.023**	**0.179**
**Education Level**	**0.100**	**0.035**	**0.004**	**0.031**	**0.168**
**Household Income**	**0.206**	**0.034**	**0.000**	**0.140**	**0.272**
Rurality	0.006	0.034	0.858	-0.060	0.073
Job Disruption	-0.002	0.036	0.946	-0.072	0.067
Survey Year	0.042	0.033	0.202	-0.022	0.105

Table S17. Linear regression results predicting the effects of foraging on daily fruit intake (cup equivalents). Statistically significant ($p<0.05$) results are bolded for emphasis.

**Fruit Intake**	**Coefficient**	**Standard Error**	**p-value**	**95% CI**	
Foraging	0.033	0.029	0.248	-0.023	0.089
Race/Ethnicity	-0.051	0.038	0.175	-0.125	0.023
**Gender Identity**	**-0.051**	**0.022**	**0.018**	**-0.093**	**-0.009**
Age	0.004	0.022	0.842	-0.038	0.047
**Education Level**	**0.043**	**0.019**	**0.027**	**0.005**	**0.080**
**Household Income**	**0.061**	**0.019**	**0.001**	**0.024**	**0.098**
Rurality	0.000	0.019	0.995	-0.037	0.038
Job Disruption	-0.004	0.020	0.858	-0.043	0.036
Survey Year	0.022	0.018	0.226	-0.014	0.058

Table S18. Linear regression results predicting the effects of foraging on daily vegetable intake (cup equivalents). Statistically significant (p<0.05) results are bolded for emphasis.

**Vegetable Intake**	**Coefficient**	**Standard Error**	**p-value**	**95% CI**	
**Foraging**	**0.078**	**0.031**	**0.012**	**0.017**	**0.138**
Race/Ethnicity	-0.018	0.034	0.601	-0.084	0.049
**Gender Identity**	**-0.203**	**0.022**	**0.000**	**-0.247**	**-0.160**
**Age**	**0.092**	**0.025**	**0.000**	**0.043**	**0.141**
**Education Level**	**0.042**	**0.021**	**0.045**	**0.001**	**0.084**
**Household Income**	**0.127**	**0.020**	**0.000**	**0.087**	**0.167**
Rurality	0.010	0.021	0.622	-0.030	0.050
Job Disruption	0.012	0.022	0.575	-0.030	0.054
Survey Year	0.021	0.020	0.281	-0.017	0.060

Table S19. Linear regression results predicting the combined effects of gardening, foraging, and preserving food on daily fruit and vegetable intake (cup equivalents). Statistically significant (p<0.05) results are bolded for emphasis.

**Fruit and Vegetable Intake**	**Coefficient**	**Standard Error**	**p-value**	**95% CI**	
**Gardening**	**0.135**	**0.037**	**0.000**	**0.064**	**0.207**
Preserving Food	0.028	0.040	0.487	-0.050	0.106
Foraging	0.045	0.056	0.421	-0.064	0.154
Race/Ethnicity	-0.045	0.058	0.438	-0.159	0.069
**Gender Identity**	**-0.251**	**0.037**	**0.000**	**-0.323**	**-0.179**
**Age**	**0.087**	**0.040**	**0.029**	**0.009**	**0.165**
**Education Level**	**0.087**	**0.035**	**0.012**	**0.019**	**0.155**
**Household Income**	**0.194**	**0.033**	**0.000**	**0.128**	**0.260**
Rurality	0.011	0.034	0.750	-0.055	0.077
Job Disruption	-0.008	0.035	0.818	-0.078	0.061
Survey Year	0.031	0.033	0.343	-0.034	0.096

Table S20. Linear regression results predicting the combined effects of gardening, foraging, and preserving food on daily fruit intake (cup equivalents). Statistically significant (p<0.05) results are bolded for emphasis.

Fruit and Vegetable Intake	Coefficient	Standard Error	p-value	95% CI	
**Gardening**	**0.058**	**0.021**	**0.005**	**0.018**	**0.099**
Preserving Food	0.029	0.023	0.212	-0.016	0.074
Foraging	0.001	0.030	0.981	-0.059	0.060
Race/Ethnicity	-0.052	0.038	0.166	-0.126	0.022
**Gender Identity**	**-0.053**	**0.021**	**0.013**	**-0.095**	**-0.011**
Age	-0.003	0.022	0.888	-0.045	0.039
Education Level	0.036	0.019	0.060	-0.002	0.074
**Household Income**	**0.055**	**0.019**	**0.004**	**0.018**	**0.093**
Rurality	0.002	0.019	0.921	-0.036	0.039
Job Disruption	-0.007	0.020	0.722	-0.046	0.032
Survey Year	0.016	0.019	0.403	-0.021	0.052

Table S21. Linear regression results predicting the combined effects of gardening, foraging, and preserving food on daily vegetable intake (cup equivalents). Statistically significant (p<0.05) results are bolded for emphasis.

Fruit and Vegetable Intake	Coefficient	Standard Error	p-value	95% CI	
Gardening	0.077	0.022	3.530	0.034	0.120
Preserving Food	0.012	0.024	0.627	-0.035	0.059
Foraging	0.044	0.034	0.190	-0.022	0.111
Race/Ethnicity	-0.018	0.034	0.601	-0.084	0.049
**Gender Identity**	**-0.206**	**0.022**	**0.000**	**-0.249**	**-0.162**
**Age**	**0.084**	**0.025**	**0.001**	**0.035**	**0.133**
Education Level	0.036	0.021	0.090	-0.006	0.077
**Household Income**	**0.120**	**0.020**	**0.000**	**0.080**	**0.160**
Rurality	0.013	0.021	0.531	-0.027	0.053
Job Disruption	0.009	0.022	0.673	-0.033	0.051
Survey Year	0.016	0.020	0.423	-0.023	0.055

Table S22. Linear regression results predicting the effects of gardening on daily combined fruit and vegetable intake (cup equivalents), by food security status. Statistically significant ($p<0.05$) results are bolded for emphasis.

**Fruit and Vegetable Intake**	**Coefficient**	**Standard Error**	**p-value**	**95% CI**	
		Food Secure			
**Gardening**	**0.120**	**0.043**	**0.006**	**0.035**	**0.205**
Race/Ethnicity	-0.114	0.084	0.175	-0.279	0.051
**Gender Identity**	**-0.222**	**0.045**	**0.000**	**-0.311**	**-0.132**
Age	0.044	0.047	0.351	-0.049	0.137
Education Level	0.057	0.045	0.203	-0.031	0.146
**Household Income**	**0.177**	**0.044**	**0.000**	**0.092**	**0.263**
Rurality	-0.060	0.043	0.165	-0.144	0.025
Job Disruption	0.081	0.047	0.087	-0.012	0.173
**Survey Year**	**0.120**	**0.043**	**0.005**	**0.037**	**0.204**
		Food Insecure			
**Gardening**	**0.220**	**0.055**	**0.000**	**0.113**	**0.327**
Race/Ethnicity	-0.049	0.076	0.519	-0.197	0.100
**Gender Identity**	**-0.337**	**0.063**	**0.000**	**-0.461**	**-0.212**
Age	0.018	0.085	0.830	-0.149	0.185
Education Level	0.054	0.061	0.372	-0.065	0.174
Household Income	0.077	0.057	0.179	-0.035	0.189
Rurality	0.099	0.057	0.081	-0.012	0.210
Job Disruption	-0.015	0.053	0.781	-0.119	0.090
Survey Year	-0.096	0.057	0.092	-0.208	0.016

Table S23. Linear regression results predicting the effects of preserving food on daily combined fruit and vegetable intake (cup equivalents), by food security status. Statistically significant (p<0.05) results are bolded for emphasis.

**Fruit and Vegetable Intake**	**Coefficient**	**Standard Error**	**p-value**	**95% CI**	
		Food Secure			
**Preservation**	**0.096**	**0.049**	**0.050**	**0.000**	**0.192**
Race/Ethnicity	-0.123	0.084	0.141	-0.287	0.041
**Gender Identity**	**-0.221**	**0.046**	**0.000**	**-0.311**	**-0.131**
Age	0.051	0.047	0.279	-0.041	0.143
Education Level	0.062	0.045	0.173	-0.027	0.151
**Household Income**	**0.186**	**0.043**	**0.000**	**0.101**	**0.271**
Rurality	-0.062	0.043	0.151	-0.147	0.023
Job Disruption	0.075	0.047	0.114	-0.018	0.168
**Survey Year**	**0.109**	**0.044**	**0.013**	**0.023**	**0.195**
		Food Insecure			
Preservation	0.070	0.060	0.245	-0.048	0.188
Race/Ethnicity	-0.039	0.076	0.603	-0.189	0.110
**Gender Identity**	**-0.348**	**0.065**	**0.000**	**-0.476**	**-0.220**
Age	-0.009	0.087	0.919	-0.179	0.161
Education Level	0.062	0.063	0.326	-0.062	0.185
Household Income	0.103	0.060	0.088	-0.015	0.221
Rurality	0.082	0.056	0.144	-0.028	0.192
Job Disruption	-0.004	0.054	0.947	-0.110	0.103
Survey Year	-0.095	0.058	0.101	-0.208	0.018

Table S24. Linear regression results predicting the effects of foraging on daily combined fruit and vegetable intake (cup equivalents), by food security status. Statistically significant (p<0.05) results are bolded for emphasis.

**Fruit and Vegetable Intake**	**Coefficient**	**Standard Error**	**p-value**	**95% CI**	
		Food Secure			
**Foraging**	**0.182**	**0.070**	**0.009**	**0.045**	**0.319**
Race/Ethnicity	-0.118	0.084	0.159	-0.283	0.047
**Gender Identity**	**-0.209**	**0.046**	**0.000**	**-0.300**	**-0.119**
Age	0.064	0.047	0.173	-0.028	0.157
Education Level	0.069	0.045	0.122	-0.019	0.157
**Household Income**	**0.180**	**0.043**	**0.000**	**0.094**	**0.265**
Rurality	-0.056	0.043	0.198	-0.141	0.029
Job Disruption	0.074	0.048	0.123	-0.020	0.168
**Survey Year**	**0.125**	**0.042**	**0.003**	**0.042**	**0.209**
		Food Insecure			
Foraging	0.015	0.080	0.851	-0.142	0.172
Race/Ethnicity	-0.037	0.075	0.620	-0.185	0.111
**Gender Identity**	**-0.342**	**0.067**	**0.000**	**-0.474**	**-0.210**
Age	-0.008	0.088	0.925	-0.182	0.165
Education Level	0.067	0.063	0.284	-0.056	0.190
Household Income	0.107	0.061	0.078	-0.012	0.226
Rurality	0.086	0.057	0.130	-0.025	0.197
Job Disruption	-0.006	0.054	0.919	-0.112	0.101
Survey Year	-0.094	0.057	0.103	-0.206	0.019

Table S25. Linear regression results predicting the effects of engagement in any HWFP activity on daily combined fruit and vegetable intake (cup equivalents), by food security status. Statistically significant (p<0.05) results are bolded for emphasis.

Fruit and Vegetable Intake	Coefficient	Standard Error	p-value	95% CI	
		Food Secure			
Any HWFP	0.079	0.044	0.074	-0.008	0.165
Race/Ethnicity	-0.123	0.085	0.147	-0.289	0.043
**Gender Identity**	**-0.222**	**0.046**	**0.000**	**-0.312**	**-0.133**
Age	0.055	0.047	0.239	-0.037	0.147
Education Level	0.062	0.045	0.173	-0.027	0.150
**Household Income**	**0.181**	**0.044**	**0.000**	**0.095**	**0.267**
Rurality	-0.059	0.043	0.171	-0.145	0.026
Job Disruption	0.084	0.047	0.077	-0.009	0.177
**Survey Year**	**0.119**	**0.043**	**0.005**	**0.035**	**0.202**
		Food Insecure			
**Any HWFP**	**0.140**	**0.050**	**0.005**	**0.041**	**0.238**
Race/Ethnicity	-0.042	0.076	0.584	-0.192	0.108
**Gender Identity**	**-0.337**	**0.064**	**0.000**	**-0.463**	**-0.211**
Age	0.014	0.088	0.872	-0.158	0.186
Education Level	0.063	0.062	0.309	-0.059	0.186
Household Income	0.086	0.059	0.144	-0.029	0.201
Rurality	0.089	0.056	0.116	-0.022	0.200
Job Disruption	-0.010	0.054	0.857	-0.115	0.096
Survey Year	-0.101	0.057	0.077	-0.214	0.011

Table S26. Logistic regression results predicting the effects of hunting on frequency of game meat intake. Statistically significant (p<0.05) results are bolded for emphasis.

Game Meat Intake Frequency	Odds Ratio	Standard Error	p-value	95% CI	
**Hunting**	**17.25**	**3.050**	**0.000**	**12.193**	**24.390**
Race/Ethnicity	0.87	0.231	0.612	0.521	1.469
**Gender Identity**	**0.52**	**0.074**	**0.000**	**0.391**	**0.683**
**Age**	**0.33**	**0.063**	**0.000**	**0.228**	**0.482**
Education Level	0.84	0.117	0.201	0.635	1.100
Household Income	0.94	0.133	0.666	0.713	1.242
Rurality	0.99	0.138	0.961	0.757	1.303
**Job Disruption**	**1.43**	**0.196**	**0.010**	**1.089**	**1.867**
Survey Year	1.16	0.157	0.279	0.888	1.512

Table S27. Ordinal logistic regression results predicting the effects of hunting on frequency of red meat intake. Statistically significant (p<0.05) results are bolded for emphasis.

Red Meat Intake Frequency	Odds Ratio	Standard Error	p-value	95% CI	
**Hunting**	**1.91**	**0.227**	**0.000**	**1.513**	**2.411**
Race/Ethnicity	0.97	0.154	0.868	0.714	1.329
**Gender Identity**	**0.70**	**0.062**	**0.000**	**0.586**	**0.832**
Age	1.04	0.106	0.693	0.852	1.272
**Education Level**	**0.80**	**0.070**	**0.011**	**0.675**	**0.951**
Household Income	1.17	0.105	0.073	0.985	1.399
Rurality	0.91	0.077	0.246	0.767	1.070
Job Disruption	0.94	0.086	0.504	0.787	1.125
Survey Year	0.98	0.082	0.832	0.835	1.157

Table S28. Ordinal logistic regression results predicting the effects of hunting on frequency of white meat intake. Statistically significant (p<0.05) results are bolded for emphasis.

White Meat Intake Frequency	Odds Ratio	Standard Error	p-value	95% CI	
**Hunting**	**1.36**	**0.159**	**0.009**	**1.080**	**1.710**
Race/Ethnicity	0.79	0.138	0.171	0.558	1.109
Gender Identity	1.08	0.095	0.361	0.912	1.288
**Age**	**0.78**	**0.074**	**0.010**	**0.653**	**0.944**
Education Level	0.95	0.084	0.561	0.799	1.129
**Household Income**	**1.74**	**0.157**	**0.000**	**1.456**	**2.075**
Rurality	1.16	0.100	0.081	0.982	1.375
**Job Disruption**	**1.25**	**0.115**	**0.016**	**1.043**	**1.497**
Survey Year	0.96	0.081	0.621	0.813	1.132

Table S29. Logistic regression results predicting the effects of hunting on game meat intake, by food security status. Statistically significant (p<0.05) results are bolded for emphasis.

**Game Meat Intake Frequency**	**Odds Ratio**	**Standard Error**	**p-value**	**95% CI**	
		Food Secure			
**Hunting**	**23.72**	**5.854**	**0.000**	**14.621**	**38.474**
Race/Ethnicity	1.17	0.553	0.738	0.464	2.954
**Gender Identity**	**0.56**	**0.120**	**0.007**	**0.368**	**0.852**
**Age**	**0.42**	**0.101**	**0.000**	**0.265**	**0.676**
Education Level	0.83	0.172	0.369	0.554	1.245
Household Income	1.21	0.260	0.381	0.792	1.841
Rurality	0.87	0.178	0.486	0.579	1.297
Job Disruption	1.16	0.261	0.521	0.742	1.800
Survey Year	1.28	0.265	0.233	0.853	1.921
		Food Insecure			
**Hunting**	**13.27**	**3.656**	**0.000**	**7.731**	**22.767**
Race/Ethnicity	0.95	0.348	0.896	0.466	1.949
**Gender Identity**	**0.43**	**0.091**	**0.000**	**0.284**	**0.650**
**Age**	**0.28**	**0.119**	**0.003**	**0.121**	**0.644**
Education Level	1.15	0.244	0.515	0.757	1.743
Household Income	1.09	0.238	0.700	0.708	1.671
Rurality	1.11	0.230	0.619	0.738	1.666
Job Disruption	1.23	0.256	0.316	0.819	1.852
Survey Year	0.98	0.200	0.920	0.657	1.461

Table S30. Ordinal logistic regression results predicting the effects of hunting on frequency of red meat intake, by food security status. Statistically significant (p<0.05) results are bolded for emphasis.

**Red Meat Intake Frequency**	**Odds Ratio**	**Standard Error**	**p-value**	**95% CI**	
		Food Secure			
**Hunting**	**1.99**	**0.327**	**0.000**	**1.446**	**2.749**
Race/Ethnicity	1.12	0.296	0.663	0.669	1.880
**Gender Identity**	**0.63**	**0.074**	**0.000**	**0.500**	**0.791**
Age	1.02	0.128	0.849	0.801	1.309
**Education Level**	**0.73**	**0.085**	**0.006**	**0.580**	**0.915**
Household Income	1.16	0.141	0.235	0.910	1.469
**Rurality**	**0.75**	**0.083**	**0.009**	**0.601**	**0.929**
Job Disruption	0.97	0.130	0.820	0.746	1.261
Survey Year	0.90	0.100	0.363	0.729	1.123
		Food Insecure			
Hunting	2.23	0.435	4.110	1.520	3.267
Race/Ethnicity	0.79	0.162	0.244	0.525	1.178
Gender Identity	0.85	0.132	0.303	0.631	1.154
Age	0.74	0.182	0.222	0.457	1.199
Education Level	0.84	0.129	0.251	0.620	1.133
Household Income	0.97	0.151	0.846	0.715	1.317
Rurality	1.24	0.179	0.128	0.939	1.650
Job Disruption	1.09	0.157	0.548	0.823	1.444
Survey Year	1.09	0.154	0.553	0.824	1.437

Table S31. Ordinal logistic regression results predicting the effects of hunting on frequency of white meat intake, by food security status. Statistically significant (p<0.05) results are bolded for emphasis.

**White Meat Intake Frequency**	**Odds Ratio**	**Standard Error**	**p-value**	**95% CI**	
		Food Secure			
Hunting	1.25	0.183	0.134	0.935	1.660
Race/Ethnicity	0.82	0.210	0.448	0.499	1.359
Gender Identity	1.15	0.134	0.238	0.913	1.442
**Age**	**0.73**	**0.085**	**0.006**	**0.577**	**0.913**
Education Level	0.85	0.101	0.168	0.673	1.071
**Household Income**	**1.56**	**0.193**	**0.000**	**1.226**	**1.989**
Rurality	1.02	0.114	0.858	0.819	1.271
**Job Disruption**	**1.49**	**0.196**	**0.003**	**1.147**	**1.925**
Survey Year	0.87	0.097	0.223	0.704	1.085
		Food Insecure			
**Hunting**	**1.75**	**0.354**	**0.006**	**1.177**	**2.602**
Race/Ethnicity	0.67	0.181	0.135	0.392	1.135
Gender Identity	0.87	0.131	0.355	0.647	1.169
**Age**	**0.61**	**0.150**	**0.043**	**0.373**	**0.983**
Education Level	1.06	0.163	0.709	0.783	1.433
**Household Income**	**1.44**	**0.238**	**0.026**	**1.045**	**1.994**
**Rurality**	**1.41**	**0.206**	**0.020**	**1.054**	**1.873**
Job Disruption	1.13	0.158	0.399	0.855	1.483
Survey Year	1.20	0.174	0.217	0.900	1.591
